# Characterization of bacterial communities in wastewater with enhanced taxonomic resolution by full-length 16S rRNA sequencing

**DOI:** 10.1038/s41598-019-46015-z

**Published:** 2019-07-04

**Authors:** Daniela Numberger, Lars Ganzert, Luca Zoccarato, Kristin Mühldorfer, Sascha Sauer, Hans-Peter Grossart, Alex D. Greenwood

**Affiliations:** 1 Leibniz Institute for Zoo and Wildlife Research, Alfred-Kowalke-Straße 17, 10315 Berlin, Germany; 2 Leibniz Institute of Freshwater Ecology and Inland Fisheries, Alte Fischerhütte 2, 16775 Stechlin, Germany; 3 GFZ German Research Centre for Geosciences, Section 5.3 Geomicrobiology, Telegrafenberg C-422, 14473 Potsdam, Germany; 4 University of Göttingen, Experimental Phycology and Culture Collection of Algae (SAG), Nikolausberger Weg 18, 37073 Göttingen, Germany; 5 Max Delbrück Center for Molecular Medicine in the Helmholtz Association (BIMSB/BIH), Robert-Rössle-Strasse 10, 13092 Berlin, Germany; 6 University of Potsdam, Institute of Biochemistry and Biology, Maulbeeralle 2, 14469 Potsdam, Germany; 7 Berlin-Brandenburg Institute of Advanced Biodiversity Research (BBIB), Altensteinstrasse 32, 14195 Berlin, Germany; 8 Freie Universität Berlin, Department of Veterinary Medicine, Institute for Virology, Robert von Ostertag-Straße 7–13, 14163 Berlin, Germany

**Keywords:** Microbial ecology, Hydrology

## Abstract

Wastewater treatment is crucial to environmental hygiene in urban environments. However, wastewater treatment plants (WWTPs) collect chemicals, organic matter, and microorganisms including pathogens and multi-resistant bacteria from various sources which may be potentially released into the environment via WWTP effluent. To better understand microbial dynamics in WWTPs, we characterized and compared the bacterial community of the inflow and effluent of a WWTP in Berlin, Germany using full-length 16S rRNA gene sequences, which allowed for species level determination in many cases and generally resolved bacterial taxa. Significantly distinct bacterial communities were identified in the wastewater inflow and effluent samples. Dominant operational taxonomic units (OTUs) varied both temporally and spatially. Disease associated bacterial groups were efficiently reduced in their relative abundance from the effluent by the WWTP treatment process, except for *Legionella* and *Leptospira* species which demonstrated an increase in relative proportion from inflow to effluent. This indicates that WWTPs, while effective against enteric bacteria, may enrich and release other potentially pathogenic bacteria into the environment. The taxonomic resolution of full-length 16S rRNA genes allows for improved characterization of potential pathogenic taxa and other harmful bacteria which is required to reliably assess health risk.

## Introduction

Drinking water is a critical resource for which it is challenging to maintain hygiene in urban areas under persistent anthropogenic influence^1–4^. Pollutants and antibiotic resistant bacteria are constantly released by wastewater treatment plants (WWTP) into the environment which can result in human and (aquatic) animal health risk^5–11^. Treated sewage is also a major source of human-derived bacteria in the urban water environment, including potential pathogens that may survive the treatment process. Sewage inflow partly reflects the bacterial community of humans^12,13^. In two WWTPs in Hong Kong (China) pathogenic bacteria such as *Clostridium perfringens*, *Legionella pneumophila* and *Mycobacterium tuberculosis* like species were found to be common^14^. Thus, the WWTP effluent may not be completely depleted of (human) pathogens and the microbiome of the effluent and its nutrients may even promote the growth and proliferation of pathogenic bacteria in the environment. For example, Wakelin *et al*.^15^ demonstrated that constant effluent input in combination with increased nutrient levels in the sediment downstream of a WWTP in Australia affected the bacterial community in the sediment substantially and increased the overall diversity. In addition, a study in rural Bangladesh revealed anthropogenic contamination of groundwater pumped from shallow tubewells with faecal bacteria from the genera *Shigella* and *Vibrio*^16^ indicating the potential risk of faecal contamination of the natural environment via anthropogenic effluents.

WWTPs are considered hotspots for antibiotic resistant genes and for the spread of bacteria into the environment^9,17^. The presence of antibiotic resistant bacteria also increases the potential risk of gene transfer to non-resistant bacteria^18–20^. Several environmental bacteria are prone to developing multidrug resistance such as *Acinetobacter* spp., *Aeromonas* spp. and *Pseudomonas* spp.^21–23^. Adapted to humid and various aquatic environments^24–31^ these human-derived bacteria are part of the microbial communities in municipal WWTPs^32–34^. For example, an increase of antibiotic resistant *Acinetobacter* spp. in WWTPs has been shown by Zhang *et al*.^21^.

In addition, bacterial communities of wastewater include members of different taxonomic, biochemical (e.g. N_2_-fixation, nitrification, denitrification, sulphur oxidation) and physiological (e.g. anaerobic, aerobic, phototrophic, heterotrophic) groups, of which many provide functional advantages for water cleaning, such as nutrient removal. However, these communities also contain bacteria of human and animal origin which may interact with the bacterial communities of natural waters (e.g. rivers and lakes) in unpredictable ways. There are few studies comparing WWTP bacterial communities in inflow and effluent with the few undertaken restricted to a few countries, i.e. the USA, Hong Kong (China), and Spain^5,12,13,35,36^. Furthermore, these studies have provided relatively little taxonomic resolution since molecular identification has been limited to short hypervariable regions of the 16S rRNA gene due to amplicon size constraints in sequencing on the Illumina or Roche 454 platforms^5,12,13,35,36^. Providing only a restricted phylogenetic resolution, these methods do not allow for the reliable identification of human pathogenic bacteria in the environment. Full 16S rRNA gene sequencing, however, can provide improved taxonomic identification on the genus and species level. Therefore, we used single molecule real time (SMRT) sequencing (PacBio® Sequel platform) to determine full-length bacterial 16S rRNA gene PCR amplicon sequences in order to improve the characterization of bacterial communities in wastewater samples and to compare the communities between inflow and effluent samples. Inflow and effluent samples from a WWTP in Berlin (Germany) were collected every three months for one year to characterize in detail and compare the bacterial communities including potentially pathogenic bacteria.

## Results

### Bacterial community composition and dominant OTUs

Using an OTU clustering cut-off of 99% sequence similarity, we were able to identify a total of 7,068 OTUs (initial data) of which 3,860 were left after rarefaction. Beta-diversity analyses were performed on a rarefied OTU-count table. The rationale was to describe the main changes in microbial community composition and the sequence coverage of our samples (ranging from 4,468 to 78,350 reads) did not allowed for a robust analysis of the “rare-biosphere” for which 0.1–0.2 M reads per sample are recommended^37^. As expected, the numbers of reads and OTUs were reduced after rarefaction (Supplementary Fig. S1). Nevertheless, the rarefied dataset was highly representative of the original non rarefied data (R = 0.99, *p*-value < 0.001) as confirmed by a Mantel correlation test based on the Bray-Curtis dissimilarity and Person coefficient.

Fingerprinting techniques have shown that the 10–50 most abundant taxa usually contribute more than 0.1–1.0% to the total cell counts. That is why, in concordance with other studies^38–40^, bacterial phyla and genera representing more than 1.0% of the total community are considered dominant taxa.

**Figure 1:**
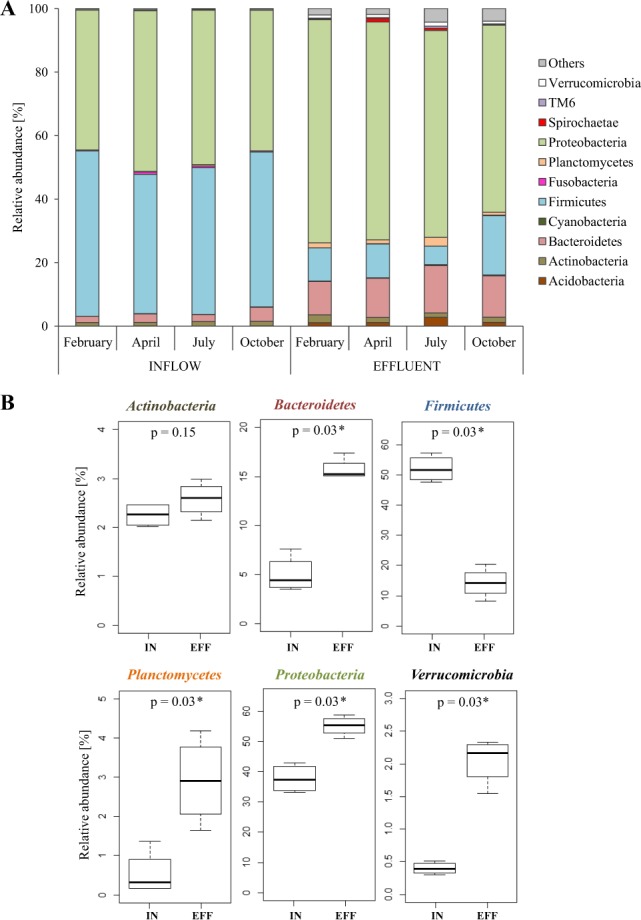
Relative abundance of dominant bacterial phyla (contributing more than 1% to the total bacterial community) in inflow and effluent samples after data rarefaction. (**A**) Bar charts show the percentage bacterial taxonomic composition for each sample. (**B**) Boxplots showing the average relative abundance of the dominant bacterial phyla between inflow and effluent. The p-value (p) indicates the significance of the differences based on a PERMANOVA with p < 0.05 being significant.

**Figure 2:**
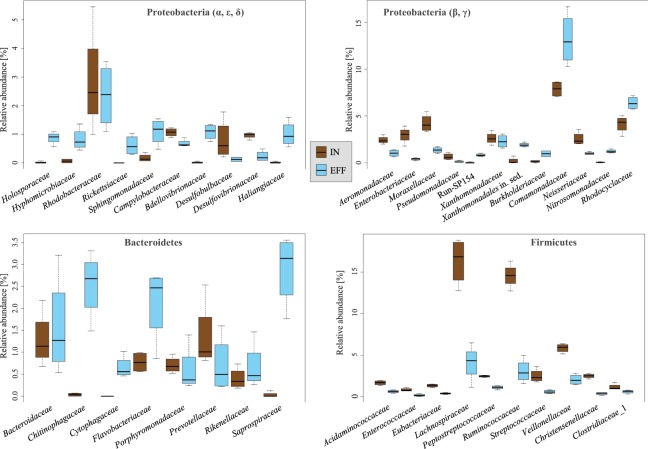
Average relative abundance of families contributing more than 1.0% to the total bacterial community in inflow and/or effluent. Only families of the three most abundant phyla Proteobacteria, Bacteroidetes and Firmicutes are shown.

Predominant phyla in the inflow were Firmicutes, Proteobacteria, Bacteroidetes and Actinobacteria with an average abundance of 52.2 ± 4.4%, 37.8 ± 4.7%, 4.9 ± 1.9%, and 2.2 ± 0.2%, respectively. In contrast, the effluent was dominated by *Proteobacteria* (54.8 ± 3.3%), Bacteroidetes (15.7 ± 1.1%), Firmicutes (14.3 ± 5.0%), Planctomycetes (2.9 ± 1.1%), Actinobacteria (2.6 ± 0.4%), Verrucomicrobia (2.1 ± 0.4%) and Acidobacteria (1.3 ± 0.4%) (Fig. 1). Families of the dominant phyla Proteobacteria, Bacteroidetes and Firmicutes contributing more than 1% in at least one sample are shown in Fig. 2. Firmicutes were dominated by *Acidaminococcaceae*, *Enterococcaceae*, *Eubacteriaceae*, *Lachnospiraceae*, *Peptostreptococcaceae*, *Ruminococcaceae*, *Streptococcaceae*, *Veillonellaceae, Christensenellacea* and *Clostridiaceae* 1 with *Lachnospiraceae* and *Ruminococcaceae* as most abundant. Proteobacteria were mainly represented by the families *Aeromonadaceae*, *Comamonadaceae*, *Enterobacteriaceae*, *Moraxellaceae*, *Neisseriaceae*, *Rhodobacteraceae*, *Rhodocyclaceae Campylobacteriaceae* and *Xanthomonadaceae*. For the phylum Bacteroidetes, the families *Bacteroidaceae*, *Chitinophagaceae*, *Cytophagaceae*, *Flavobacteriaceae*, *Porphyromonadaceae, Prevotellaceae*, *Rikenellaceae* and *Saprospiraceae* were dominant.

OTUs contributing more than 1% to the total bacterial community in at least one sample are listed in Table 1. In addition, OTUs that significantly differ between inflow and effluent are marked in Table 1 and belong to multiple genera: *Canditadus* Accumulibacter, *Candidatus* Competibacter, *Comamonadaceae* unclassified, *Dechloromonas, Nitrosomonas, Nitrospira, Paracoccus, Rhodoferax*, unclassified Run-SP154, *Simplicispira, Streptococcus*, unclassified *Saprospiraceae* and *Uruburuella*. Using full-length 16S rRNA reads, we were able to reliably identify many OTUs at high taxonomic resolution (often at species level) by comparing them with reference sequences from known bacterial species (Table 1, Table 2) based on a global SILVA alignment (SINA Aligner) for rRNA genes^41^.

**Table 1:** Relative abundance of dominant OTUs (after rarefaction) with phylogenetic affiliation of inflow and effluent samples based on the global SILVA alignment (SINA Aligner) for rRNA genes^41^.

	INFLOW				EFFLUENT				Genus	Affiliated species	Sequence Identity
	Feb	Apr	Jul	Oct	Feb	Apr	Jul	Oct			
OTU000121	0.92	0.78	2.26	1.37	0.04	0.09	0.13	0.27	*Acetoanaerobium* ↓	—	
OTU000039	4.23	2.53	3.07	5.46	0.51	0.13	1.03	1.19	*Acidovorax* ↓	—	
OTU000062	4.32	3.00	1.66	2.35	0.47	0.36	0.18	0.78	*Acidovorax* ↓	*A. defluvii* (Y18616.1)	99.5%
OTU000055	6.58	3.27	2.60	2.53	0.25	0.98	0.67	0.63	*Acinetobacter* ↓	—	
OTU000065	2.39	4.43	1.30	1.63	0.34	0.40	0.63	0.76	*Aeromonas* ↓	*A. media* (X60410.1)	97.8%
OTU000112	1.75	0.72	1.79	1.57	0.20	0.45	0.40	0.94	*Anaerosinus* ↓	*A. glycerini* (AJ010960.1)	98.5%
OTU000414	0.00	0.00	0.00	0.00	0.78	1.25	1.59	1.30	*Aquabacterium* ↑	—	
OTU000015	2.55	8.28	3.20	8.42	0.90	1.14	0.90	3.69	*Arcobacter* ↓	*A. cryaerophilus* (FR682113.1)	99.9%
OTU000307	0.45	0.07	0.34	0.51	0.18	0.40	0.11	1.10	*Bacteroides*	*B. graminisolvens* (AB547643.1)	99.8%
**OTU000237**	**0.00**	**0.02**	**0.00**	**0.00**	**1.37**	**0.58**	**1.30**	**1.70**	***Ca*** *.* **Accumulibacter ↑**	—	
**OTU000163**	**0.00**	**0.13**	**0.00**	**0.00**	**5.39**	**15.38**	**1.03**	**1.07**	***Ca*** *.* **Competibacter ↑**	—	
OTU000253	0.00	0.00	0.00	0.00	0.98	1.14	0.07	0.02	*Ca*. Nitrotoga ↑	*Candidatus* N. arctica (DQ839562.1)	99.5%
OTU001077	0.00	0.00	0.00	0.00	0.00	0.00	1.63	0.02	*Chryseobacterium* ↑	—	
OTU000006	0.00	0.00	0.00	0.00	3.04	1.19	0.16	1.95	*Comamonadaceae* uncl. ↑	—	
OTU000040	0.00	0.07	0.00	0.00	6.20	2.26	0.11	0.78	*Comamonadaceae* uncl. ↑	—	
**OTU000071**	**0.00**	**0.00**	**0.00**	**0.00**	**2.01**	**1.05**	**1.16**	**0.74**	***Comamonadaceae*** **uncl. ↑**	—	
OTU000188	0.65	0.87	1.01	0.96	0.00	0.02	0.02	0.09	*Comamonadaceae* uncl. ↓	—	
**OTU000585**	**0.00**	**0.00**	**0.00**	**0.00**	**1.12**	**0.22**	**0.16**	**0.04**	***Comamonadaceae*** **uncl. ↑**	—	
OTU000031	4.90	2.75	6.11	5.55	0.43	0.27	0.16	1.54	*Comamonas* ↓	*C. denitrificans* (AF233880.1)	99.9%
OTU000377	0.00	0.00	0.00	0.00	0.00	0.00	2.89	1.39	*Cupriavidus* ↑	—	
**OTU000210**	**0.00**	**0.02**	**0.00**	**0.00**	**1.43**	**2.04**	**0.56**	**0.51**	***Dechloromonas*** ** ↑**	—	
OTU000187	1.45	1.14	0.34	1.05	0.07	0.07	0.02	0.11	*Enterococcus* ↓	*E. aquimarinus* (EF204323.1)	99.7%
OTU000153	1.14	1.07	0.25	1.54	0.00	0.07	0.00	0.04	*Faecalibacterium* ↓	*F. prausnitzii* (LQ500116.1)	99.7%
OTU000381	0.00	0.00	0.00	0.00	0.69	0.72	1.63	0.74	*Geothrix* ↑	*G. fermentans* (U41563.1)	97.8%
OTU000133	1.81	1.43	0.92	1.34	0.09	0.20	0.02	0.29	*Lachnospiraceae* uncl. ↓	—	
**OTU000513**	**0.00**	**0.00**	**0.02**	**0.00**	**0.54**	**0.72**	**0.58**	**1.23**	***Nitrosomonas*** ** ↑**	—	
**OTU000519**	**0.00**	**0.00**	**0.00**	**0.00**	**0.22**	**0.13**	**1.19**	**1.05**	***Nitrospira*** ↑	—	
OTU000766	0.00	0.00	0.00	0.00	0.09	0.18	1.28	0.34	OM27 clade ↑	—	
**OTU000467**	**0.34**	**0.60**	**1.75**	**0.09**	**0.02**	**0.00**	**0.02**	**0.00**	***Paracoccus*** ** ↓**	***P. lutimaris*** **(KJ451483.1)**	**99.2%**
OTU000209	0.22	0.45	0.25	0.81	1.05	0.40	0.60	1.19	*Peptostreptococcaceae* uncl. ↑	—	
OTU000109	2.55	1.81	2.53	0.83	0.25	0.47	0.13	0.56	*Proteocatella* ↓	—	
**OTU000466**	**0.00**	**0.00**	**0.00**	**0.00**	**4.23**	**0.72**	**0.02**	**0.16**	***Rhodoferax*** ** ↑**	—	
**OTU000339**	**0.00**	**0.02**	**0.00**	**0.00**	**0.45**	**0.27**	**1.23**	**1.59**	**Run-SP154 uncl. ↑**	—	
**OTU000756**	**0.00**	**0.00**	**0.00**	**0.00**	**2.13**	**0.78**	**0.47**	**0.13**	***Simplicispira*** ** ↑**	***S. limi*** **(LC177120.1)**	**98.1%**
OTU000123	0.51	0.96	5.35	0.78	0.07	0.04	0.09	0.07	*Streptococcus* ↓	*S. parasuis* (AB936273.1)	97.7%
OTU000387	0.11	0.11	2.22	0.27	0.04	0.04	0.02	0.04	*Streptococcus* ↓	—	
**OTU000685**	**0.04**	**0.04**	**1.48**	**0.11**	**0.00**	**0.00**	**0.00**	**0.00**	***Streptococcus*** ** ↓**	—	
OTU000873	0.02	0.00	1.12	0.00	0.02	0.00	0.02	0.02	*Streptococcus* ↓	—	
OTU000128	1.84	1.50	0.74	1.10	0.38	0.47	0.11	0.47	*Subdoligranulum* ↓	—	
OTU000165	1.16	1.05	0.45	0.96	0.22	0.18	0.09	0.47	*Subdoligranulum* ↓	—	
OTU000078	0.27	1.28	1.84	1.03	0.92	1.16	4.07	1.88	*Thauera* ↑	—	
OTU000017	9.65	6.51	2.17	6.78	1.10	1.01	0.11	1.28	*Trichococcus* ↓	*T. flocculiformis* (JF505981.1)	99.7%
**OTU000098**	**0.00**	**0.04**	**0.00**	**0.00**	**0.78**	**0.85**	**1.19**	**0.67**	***Saprospiraceae*** **uncl. ↑**	—	
OTU001461	0.00	0.00	0.00	0.00	0.40	1.68	0.02	0.00	*Neisseriaceae* uncl. ↑	—	
**OTU000099**	**2.57**	**2.71**	**3.02**	**0.94**	**0.13**	**0.07**	**0.02**	**0.07**	***Uruburuella*** ** ↓**	***U. suis*** **(AJ586614.1)**	**99.9%**
OTU000397	0.38	0.29	1.37	0.11	0.02	0.02	0.02	0.11	*Veillonella* ↓	—	
OTU000829	0.00	0.04	0.00	0.00	0.76	1.05	0.78	0.18	*Zoogloea* ↑	—	
Sum	52.8	48.0	49.2	48.1	40.3	40.7	28.6	33.2			

Only OTUs contributing at least in one sample more than 1.0% are shown for the samples collected in February (Feb), April (Apr), July (Jul) and October (Oct). OTUs that significantly change in abundance between inflow and effluent are indicated in bold (all are highly significant with p-values < 0.001). Arrows indicate an increase (↑) or decrease (↓) from inflow to effluent.

**Table 2:** Affiliation of OTUs from potentially harmful bacterial genera and their presence in the inflow (IN) and effluent (EFF).

OTU	Presence	Genus	Affiliated species	Pairwise sequence identity	Node support (bootstrap value)
OTU004330	IN, EFF	*Aeromonas*	*A. sharmana* (KC469704.1)	99.6%	97.3
OTU001375	IN, EFF		*A. sobria* (X60412.1)	99.0%	96.9
OTU027282	EFF		*A. jandaei* (X60413.1)	99.0%	87.4
OTU000640	IN, EFF		*A. australiensis* (HE611955.1)	99.2%	79.4
OTU000065	IN, EFF		*A. media* (X60410.1)	99.7%	100
OTU014486	IN	*Acinetobacter*	*A. beijerinckii* (KU308266.1)	99.7%	98.7
OTU017554	IN		*A. schindleri* (AJ278311.1)	97.9%	98.7
OTU025938	IN, EFF		*A. haemolyticus* (AY047216.1)	99.1%	77.5
OTU006694	IN		*A. celticus* (MBDL01000001.1)	99.7%	100
OTU004317	IN, EFF		*A. lwoffii* (KT369856.1)	98.6%	95.3
OTU012127	IN		*A. albensis* (KR611794.1)	98.7%	100
OTU004358	IN		*A. harbinensis* (KC843488.1)	98.6%	95.9
OTU011363	IN		*A. ursingii* (APQC01000001.1)	99.6%	100
OTU014283	IN		*A. oleivorans* (KF749341.1)	98.0%	78.0
OTU020140	IN		*A. radioresistens* (AM495259.1)	99.1%	100
OTU017557	IN		*A. rudis* (EF204258.3)	99.3%	100
OTU012742	IN		*A. baumannii* (X81660.1)	98.6%	100
OTU025639	IN		*A. indicus* (LC191521.1)	99.1%	88.2
OTU025642	IN		*A. gerneri* (APPN01000079.1)	99.4%	100
OTU020183	IN		*A. gandensis* (KM454858.1)	99.6%	100
OTU003228	IN, EFF		*A. junii* (KJ620866.1)	99.4%	100
OTU027605	IN	*Clostridium*	*C. frigidicarnis* (AF069742.1)	98.2%	100
OTU004478	IN, EFF		*C. perfringens* (AB610566.1)	99.7%	100
OTU034705	EFF		*C. colicanis* (AJ420008.1)	98.3%	100
OTU019193	IN		*C. paraputrificum* (AB627079.1)	98.5%	91.1
OTU022098	IN, EFF		*C. disporicum* (DQ855943.1)	99.6%	94.1
OTU024338	IN		*C. botulinum* type F (X68171.1)	99.5%	85.0
OTU037193	IN		*C. butyricum* (KY203641.1)	99.0%	100
OTU005080	IN, EFF		*C. beijerinckii* (LC071788.1)	99.6%	87.9
OTU014508	IN, EFF		*C. puniceum* (X71857.1)	98.1%	81.2
OTU009104	IN, EFF	*Legionella*	*L. feeleii* (LBHK01000101.1)	99.4%	100
OTU020580	EFF		*L. lytica* (X66835.1)	99.3%	100
OTU014279	IN	*Leptospira*	*L. alstonii* (CP015217.1)	99.8%	96.1
OTU006502	EFF	*Pseudomonas*	*P. pohangensis* (DQ339144.1)	98.0%	97.8
OTU001532	IN, EFF		*P. pseudoalcaligenes* (AJ628163.1)	98.6%	100
OTU030991	IN, EFF		*P. guangdongensis* (LT629780.1)	99.6%	100
OTU001564	IN, EFF		*P. alcaligenes* (CP014784.1)	99.8%	100
OTU017978	IN		*P. aeruginosa* (DQ641680.1)	99.6%	100
OTU025592	EFF		*P. psychrotolerans* (AJ575816.1)	99.0%	100
OTU020427	IN		*P. kunmingensis* (JQ246444.1)	98.5%	100
OTU032410	IN		*P. monteilii* (AF064458.1)	98.8%	82.9
OTU001416	IN, EFF		*P. baetica* (FM201274.1)	99.6%	99.5
OTU002122	IN		*P. gessardii* (KJ589457.1)	98.8%	86.1
OTU000948	IN, EFF		*P. palleroniana* (FNUA01000001.1)	98.8%	76.2
OTU007640	IN	*Yersinia*	*Y. massiliensis (EF179119.1)*	99.3%	98.9
OTU036842	IN		*Y. frederiksenii (AF366379.1)*	99.3%	99.9
OTU036840	IN		*Y. enterocolitica (CHYV01000006.1)*	99.3%	100
OTU006891	IN		*Y. intermedia (JX429054.1)*	99.5%	94.8

This is based on the global SILVA alignment (SINA Aligner) for rRNA genes^41^ (pre rarefaction).

A comparison of the PacBio generated OTU sequences with short-read sequences, which were generated by extracting a 477 bp fragment of the hypervariable regions V3-V4 according to the primer pair of Klindworth *et al*.^42^ from the PacBio reads, was performed for the genus *Acinetobacter* as an example. Out of 113 OTUs related to the genus *Acinetobacter* 18 could be resolved to the species level when using full-length PacBio reads and 10 when using the hypervariable region V3-V4. The bootstrap values were always lower when using shorter sequences. Two OTUs yielded different phylogenetic results depending on sequence length (Supplementary Table S1, Fig. S2).

### Inflow versus effluent samples and dominant OTUs

**Figure 3:**
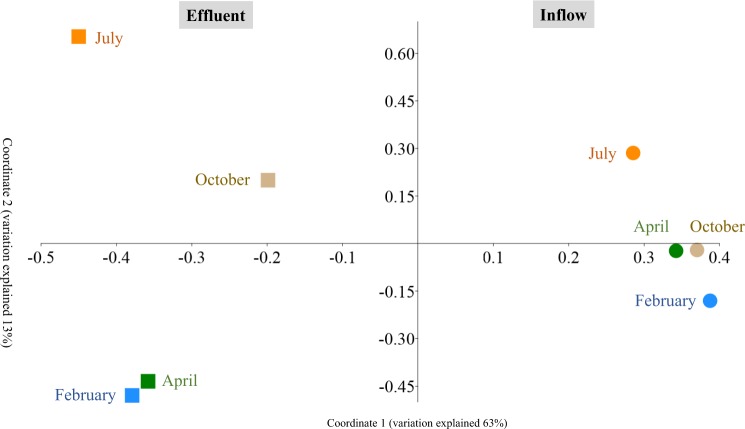
Principal coordinate analysis (PCoA) of the bacterial community based on Bray-Curtis similarity. Inflow and effluent samples are defined as circles and squares, respectively. Different sampling time points are indicated by blue colour for February, green colour for April, red colour for July and brown colour for October.

Principal coordinate analysis defined two main clusters: inflow and effluent samples (Fig. 3). Within the inflow cluster the samples from April and October were most similar to each other, whereas in the effluent cluster February and April or July and October samples were clustered more closely together. A permutational multivariate analysis of variance (PERMANOVA) revealed a significant difference between inflow and effluent samples with a p-value of 0.02. Furthermore, the dominant bacterial phyla Bacteroidetes, Firmicutes, Planctomycetes and Verrucomicrobia differed significantly in their relative abundance between inflow and effluent samples, whereas the phylum Actinobacteria did not show a significant difference between both sample groups (Fig. 1).

### Phylogenetic analysis of genera that contain known pathogens

The advantage of full-length 16S rRNA gene sequencing was that, with some restrictions, more refined and reliable taxonomic assignment, even to the species level, was possible. While most of the previous studies used only the information of certain hypervariable regions of the 16S rRNA, we were able to use all phylogenetically relevant sites of the whole 16S rRNA gene. We attempted to identify OTUs to a higher taxonomic level (e.g. species level), focusing on bacterial groups known to contain strains relevant for human health (Table 2). The analysis was carried out using maximum likelihood based phylogenetic approaches and including reference sequences from the SILVA database^43,44^. Three major groups of OTUs were identified representing (1) waterborne/-transmitted bacteria (i.e., *Legionella, Leptospira, Vibrio and Mycobacterium*)^45–48^, (2) enteric bacteria (i.e., *Campylobacter*, *Clostridium, Salmonella, Shigella and Yersinia*)^49–54^, and (3) environmental bacteria (i.e. *Acinetobacter, Aeromonas and Pseudomonas*) that include important nosocomial pathogens, which can also acquire multi-drug resistance^55–59^.

#### Waterborne bacteria: *Legionella*, *Leptospira*, *Mycobacterium* and *Vibrio*

**Figure 4:**
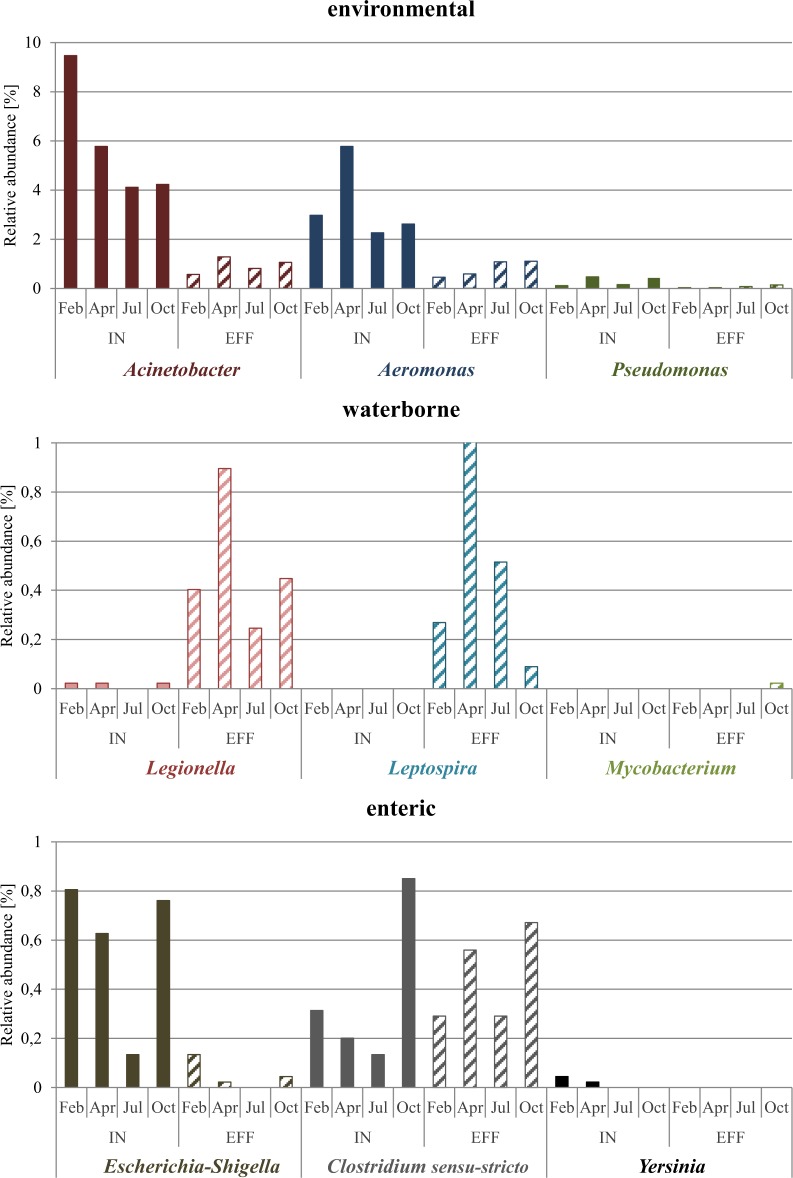
Relative abundance (after rarefaction) of genera with known potential pathogens. They were grouped in environmental, waterborne and enteric, and are shown for each sample of inflow and effluent.

*Legionella* spp. and *Leptospira* spp. contributed to up to 0.9% and 1.0% to the bacterial community after rarefaction, respectively with increasing numbers from inflow to effluent (Fig. 4). Identified OTUs were closely related to *Legionella lytica*, *L. feeleii* (Supplementary Fig. S3) and *Leptospira alstonii* **(**Supplementary Fig. S4**)**. The genus *Mycobacterium* was only present in the October effluent samples with a relative abundance of 0.02%, whereas *Vibrio* was not detected in either the inflow or the effluent (Fig. 4).

#### Enteric bacteria: *Campylobacter*, *Clostridium*, *Escherichia/Shigella*, *Salmonella* and *Yersinia*

*Campylobacter* and *Salmonella* spp. were not detectable. The genus *Clostridium* (*sensu-stricto*) contributed between 0.1–0.9% to the bacterial community. *Escherichia*/*Shigella* and *Yersinia* decreased from inflow to effluent in relative abundance with *Yersinia* spp. being absent from the effluent samples (Fig. 4). According to our phylogenetic analyses probable species are *Clostridium perfringens*, *C. botulinum*, *C. butyricum* (Supplementary Fig. S5), *Yersinia massiliensis*, *Y. frederiksenii*, *Y. enterocolitica* and *Y. media* (Supplementary Fig. S6). OTUs from the *Escherichia*/*Shigella* group did not show clear sequence similarity with any known species.

#### Environmental bacteria: *Acinetobacter*, *Aeromonas* and *Pseudomonas*

The genera *Acinetobacter, Aeromonas* and *Pseudomonas* were present in all samples, but their relative abundance decreased from inflow water to effluent in each of the sampled months (Fig. 4). *Acinetobacter* and *Aeromonas* spp. represented up to 9.5% and 5.8% of the bacterial community in the inflow, but only up to 1.3% and 1.1% in the effluent, respectively, while *Pseudomonas* spp. contributed only between 0.02% and 0.5% to the total bacterial community decreasing from inflow to effluent. OTUs were closely related to the described species *Acinetobacter beijerinckii*, *A. haemolyticus*, *A. baumannii* (Supplementary Fig. S7), *Aeromonas sharmana*, *A. media* (Supplementary Fig. S8), *Pseudomonas alcaligenes* and *P. aeruginosa* (Supplementary Fig. S9).

## Discussion

The advantage of full-length 16S rRNA gene sequencing was that, with some restrictions, more refined and reliable taxonomic assignment, even to the species level, was possible. While most of the previous studies used only the information of certain hypervariable regions of the 16S rRNA, we were able to use all phylogenetically relevant sites of the whole 16S rRNA gene. Huse *et al*.^60^ compared full-length sequence with V3 and V6 hypervariable regions and found both methods could resolve the taxonomy similarly at the level of genus. Our genus comparison of *Acinetobacter* demonstrated better species level resolution and higher phylogenetic tree node support than when sequences were restricted to the V3-V4 regions (Supplementary Table S1, Fig. S2). However, further direct experimental comparisons using amplicons from the same samples and broadening the number of taxa examined will be necessary to tell whether the advantages we observed for *Acinetobacter* are generalizable. In addition, shorter read sequencing at higher sequencing depth provides better characterization of rarer bacterial groups which is currently cost prohibitive in general at such depth with the PacBio or other long read sequencing platforms.

Few studies describing the bacterial community in inflow water compared to effluent from a WWTP based on sequence data have been performed despite the potential for contamination of water bodies in highly urbanized areas^5,12,13,35,36^. Most studies have focused on specific bacterial groups or sampled only inflow water, activated sludge or the effluent. We found distinct compositional differences between the microbiomes of WWTP inflow water and effluent using a whole 16S rRNA gene sequencing approach.

At the phylum level there were two distinct clusters based on inflow and effluent specific bacterial communities, which showed only minor temporal differences. Abiotic parameters such as oxygen concentration as well as competition among different bacterial species with different metabolic characteristics are very likely responsible for the observed differences in bacterial community composition in the WWTP inflow vs. the effluent. At the OTU level, however, there is evidence for seasonal or temporal differences (Table 1), but with only four time points sampled we could not draw any strong conclusions regarding seasonality.

While at the phylum level only minor differences occur between geographically distributed WWTPs, they differ strongly in the composition of the most abundant genera^5,12,35,36^. For example, our inflow samples shared seven dominant genera with the inflow water of a WWTP in Wisconsin (USA)^36^ and nine^12^ or three^35^ genera with a WWTP in Hong Kong (China). The genera *Acinetobacter* and *Arcobacter* were dominant in all studies and are likely common members of WWTPs worldwide^5,12,35,36^.

The differences could be further explained by other environmental parameters such as pH, temperature and salinity. The WWTP in Hong Kong, for example, treated wastewater has a salinity of 1.2% since it contains ca. 30% seawater used for the toilet flushing system in Hong Kong^35^. This may possibly favour other bacterial groups in comparison to WWTPs that treat freshwater. Other reasons for the contrasting results might be the use of different small pore size filters for collecting bacteria and the application of different DNA extraction and sequencing methods. While part of the WWTP bacterial community reflects the human microbiome^13,61,62^, some bacteria likely stem from industrial waste. Environmental bacteria may reach the WWTP via rainfall and wildlife such as rodents inhabiting the drainage system. This might also explain observed regional differences in the bacterial community of WWTPs.

The dominant bacteria found in the current study can be useful or even necessary for the treatment process. *Comamonas denitrificans* has been shown to be a key organism in WWTPs and thus is very useful by its efficient denitrifying activity^63,64^. Its higher abundance in the outflow samples agreed with its presence in biofilms of the WWTP facility itself, including activated sludge^63–66^. Other species have been identified as abundant members in activated sludge and were suggested to be involved in nutrient removal including nitrite oxidation by *Nitrospira* spp. or enhanced biological phosphorus removal by *Simplicispira limi*^67–71^, which were also abundant in the effluent of the current study.

Bacteria can be harmful for humans and animals by being pathogenic and/or by carrying antibiotic resistance genes. We grouped bacterial genera that contain known pathogenic species into three categories: waterborne, enteric or environmental bacteria that are prone to multidrug resistances. Waterborne bacteria can live in water and use water as vector to spread infection^45^. Enteric pathogens normally live in the intestines of humans or animals and cause gastrointestinal disease^49,50^. Transmission of enteric pathogens occurs mainly via the fecal-oral route and contaminated water can serve as a potential vector. Among environmental bacteria are multi-antimicrobial resistant species and species which are potentially pathogenic^56,57^.

Among **waterborne** bacteria *Vibrio cholera* is a well-studied waterborne pathogen^46,72^ and has been found in WWTPs in Hong Kong, South Africa, USA and Brazil^14,73–75^. Contamination of WWTPs by cholera bacteria is likely human patient derived. As the incidence of cholera in Germany is negligible, this would explain why we never detected OTUs related to the genus *Vibrio*. *Legionella* and *Leptospira*, two other classical waterborne bacterial genera comprise known pathogenic species such as *Legionella pneumophila* and *Leptospira interrogans*. The relative abundance of OTUs belonging to these two genera increased from inflow to effluent samples indicating a potential health risk due to contamination of the environment or infection risk for WWTP workers. *Legionella* spp. are intracellular parasites and can replicate in free-living amoebae^76,77^. They likely form biofilms in the WWTP, which can promote bacterial growth and persistence in the aquatic environment^76,77^. In the current study, *L. lytica* and *L. feeleii* were identified as closest relatives (Supplementary Fig. S3). While the OTU related to *L. lytica* is exclusively present in the inflow samples, the OTU related to *L. feeleii* was detected in both inflow and effluent samples. Both species are known to cause pneumonia in humans when inhaled via aerosols^78–81^ and may present a potential health risk as *Legionellae* in WWTP aerosols are not unusual^82–84^. Wastewater, being enriched in nutrients and carbon, dissolved oxygen concentrations of 6.3–10.3 mg/L, and relatively high temperatures of 14.5–24.6 °C (Supplementary Fig. S10), provides favourable conditions for replication of *Legionella* spp.^85–87^. These pathogens remain challenging to control as they grow successfully within protozoa and biofilms, where they are relatively protected against disinfectants, grazers and other harsh environmental conditions^85^.

The increase of *Leptospira* in the wastewater effluent could be associated with the presence of saprophytic leptospires that reproduce outside of a host and inhabit various aquatic environments^88,89^. Pathogenic *Leptospira*, however, can survive in water but do not reproduce outside of a host and thus may be introduced via an infected person or animals such as rodents, which are their natural reservoir and shed leptospires into their environment via urine^88,89^. Our phylogenetic analyses showed that the OTU affiliated with *L. alstonii* clustered with known pathogenic species such as *L. interrogans* and *L. mayottensis*^90,91^ and was only present with one read in one of the inflow samples and thus, is likely derived from an infected human or rodent. All other *Leptospira* OTUs, were exclusively present in the effluent samples and belonged to saprophytic species such as *L. idonii* and *L. biflexa*^92,93^ or were represented by their own cluster (Supplementary Fig. S4). This indicates that wastewater might favour the persistence or possible growth of saprophytic leptospires. While pathogenic leptospires grow much better at temperatures of around 30 °C, saprophytic *Leptospira* spp. also replicate well at lower temperatures, as low as 10 °C^94^. The temperatures of our wastewater samples varied between 14.5–24.6 °C during the sampled year (Supplementary Fig. S10). Furthermore, the ability to form biofilms may enhance their survival and/or replication in such an environment. However, as most of these OTUs were related to saprophytic leptospires, we would assume a low health risk potential for humans and animals.

**Enteric** pathogens can secrete (entero-) toxins, which can damage the gastrointestinal tract of infected individuals^95–97^. They are part of the excreted faecal microbiota of humans in the WWTP inflow, but can also be introduced by animals such as rodents^98^. In the current study, *Clostridium* (sensu-stricto), *Escherichia*-*Shigella*, and *Yersinia* were mainly not abundant in the inflow, having a maximum relative abundance of 0.9% and were reduced in or absent from the effluent (Fig. 4). *Campylobacter* and *Salmonella* spp. were not detected at all, which could mean that the sequencing depth was too low to detect them. While *Escherichia*-*Shigella* and *Yersinia* decreased  in relative abundance, *Clostridium* (sensu-stricto) remained mainly stable as observed previously^12^. These findings indicate that the wastewater treatment works well in removing enteric bacteria by introducing oxygen, preventing serious health risk.

**Environmental** bacteria such as *Acinetobacter*, *Aeromonas* and *Pseudomonas* spp. can be multidrug resistant^55,99,100^ and some species also have a pathogenic potential such as *Pseudomonas aeruginosa* and *Acinetobacter baumannii*^101,102^. OTUs related to species like *P. aeruginosa* and *A. baumannii* were not abundant and only present in the WWTP inflow suggesting that the treatment procedures are effective against these species. Although the overall relative abundance of these three genera was reduced, they were not completely removed during the treatment process.

Pathogens can be strongly diluted in wastewater samples and masked by other much more abundant bacteria. Thus, the presence of pathogens could be greatly underestimated when using 16S rRNA data only. For instance, in a previous study we could detect and isolate *C. difficile* from the same samples used in the current study and even detect the *C. difficile* toxin genes via quantitative real-time PCR, but the 16S rRNA dataset did not provide any evidence for the presence of *C. difficile*^103^. Therefore, there are clearly limits to high throughput sequencing studies that involve a PCR step in terms of favouring abundant taxa. According to Huse *et al*.^60^ sequencing of a hypervariable region covers higher bacterial diversity in comparison to full-length sequencing as a consequence of higher sequencing depth. However, the information provided by full-length 16S rRNA enhances species identification and taxonomic resolution including for potential pathogens. Thus, the choice of the sequencing approach will be based whether less abundant taxa detection or taxonomic resolution are more critical to a given study. In addition, 16S rRNA amplicon data do not reflect absolute abundance of bacteria due to PCR amplification steps and considering the variability in 16S rRNA gene copy numbers among different bacterial taxa^104,105^. Thus, changes in relative abundances in the current study represent changes in the proportion of bacterial groups between the samples and not absolute quantitative differences. Consequently, an increase in relative abundance (i.e. *Legionella* or *Leptospira*) does not necessarily represent an increase in absolute abundance (e.g. growth). The number of bacterial cells in WWTP effluent are usually up to two orders of magnitude lower than in the inflow^106,107^ This means that an increase of the relative abundance or proportion of *Legionella* and *Leptospira* in the effluent does not necessarily reflect an increase in absolute abundance (cell numbers). However, we hypothesize that the efficiency of the wastewater treatment removal capability of such potential pathogenic taxa is lower in comparison to enteric bacteria.

The current study provided evidence for the presence of potential pathogens such as *Acinetobacter baumannii*, *Clostridium perfringens*, *Legionella lytica, Pseudomonas aeruginosa* and *Yersinia enterocolitica* from the full-length 16S rRNA gene, which may indicate that they are much more abundant than *C. difficile*, although still rare in the 16S rRNA dataset. Thus, further studies including isolation and cultivation methods are necessary to further investigate the presence and diversity of pathogens, to test for infectivity and to assess a realistic health risk. Water-adapted pathogens, in particularly, such as within the genus *Legionella* or *Leptospira* potentially increase in WWTPs and hence should be of great interest for health risk assessment, WWTP operation and waste management.

## Material and Methods

### Sampling

Untreated raw inflow water and treated effluent of a wastewater treatment plant (WWTP) in Berlin, Germany, were sampled four times in 2016 (February 11^th^, April 15^th^, July 27^th^ and October 20^th^). The sampled effluent had no contact with environment and was already disinfected representing effluent that goes to the environment. The selected WWTP treated municipal wastewater with only a minor percentage of industrial wastewater. It contains a mechanical treatment followed by biological one which includes biological phosphate elimination in combination with nitrification and denitrification, and the production of activated sludge. The effluent undergoes UV sterilization before its release in the environment. The exact location of the sampled WWTP cannot be disclosed due to a confidentiality agreement with the WWTP operators. The water samples were filtered through 0.22 µm Sterivex® filters (EMD Millipore, Germany) connected to a peristaltic pump (EMD Millipore, Germany) to concentrate bacteria and subsequently stored at −20 °C. From the inflow water 20–35 mL could be concentrated on one filter, while from the effluent it was possible to filtrate 175–500 mL. Temperature, pH and dissolved oxygen were measured in the inflow samples with a digital thermometer (Carl Roth, Germany), pH multimeter EC8 (OCS.tec GmbH & CO. KG, Germany), Pen type, IP 67 dissolved oxygen meter (PDO-519, Lutron Electronic Enterprise CO., Taiwan), respectively.

### DNA extraction

DNA was extracted from 0.22 µm Sterivex filters using the QIAamp DNA mini kit (Qiagen, Germany) following the protocol for tissue with some modifications. Briefly, the filters were cut into pieces and put into a 2 mL tube. 0.2 µm zirconium glass beads and 360 µL of buffer ATL were added and vortexed for 5 min at 3,000 rpm in an Eppendorf MixMate® (Eppendorf, Germany). Proteinase K (>600 mAU/ml, 40 µL) was added and incubated at 57 °C for 1 h. After centrifugation for 1 min at 11,000 rpm, the supernatant was transferred to a new 2 mL tube and extraction was performed following the manufacturer’s protocol.

### Amplification of full-length 16S rRNA genes

Primers 27F (5′-AGRGTTYGATYMTGGCTCAG-3′) and 1492R (5′-RGYTACCTTGTTACGACTT-3′) were used with symmetric barcodes designed by Pacific Biosciences® (USA) for each sample. PCRs for each sample were run in triplicate and carried out in a total volume of 25 µL containing 12.5 µL MyFi^TM^ Mix (Bioline, UK), 9.3 µL water, 0.7 µL of bovine serum albumin (20 mg/mL; New England Biolabs, USA), 0.75 µL of each primer (10 µM) and 1 µL of DNA. The cycling program was as follows: denaturation at 95 °C for 3 min, 25 cycles of 95 °C for 30 sec, 57 °C for 30 sec and 72 °C for 60 sec and a final elongation at 72 °C for 3 min. The quality and concentration of the PCR products were determined using a 4200 TapeStation with D5000 tapes and reagents (Agilent Technologies, USA). Equimolar sample mixes were used for library preparation. Three negative controls were included containing 1 µL water instead of DNA resulting in 13, 13 and 123 total reads representing 5, 8 and 14 OTUs, respectively. OTUs with a read number >5 (=three OTUs in total) in the negative controls were related to the genera *Aquabacterium*, *Ralstonia* and *Pelomonas*, which were subsequently removed from the whole data set prior to analysis.

### Library preparation and sequencing

After bead purification with Agencourt AMPure XP (Beckman Coulter, USA), sequencing libraries were built using the SMRTbell Template Prep Kit 1.0‐SPv3 following the guidelines in the amplicon template protocol (Pacific Biosciences, USA). DNA damage repair, end-repair and ligation of hairpin adapters were performed according to the manufacturer’s instruction. DNA template libraries were bound to the Sequel polymerase 2.0 using the Sequel Binding Kit 2.0 (Pacific Biosciences, USA). The data collection per sample was done in a single Sequel SMRT Cell 1M v2 with 600 min movie time on the Sequel system (Pacific Biosciences, USA). We used a 5 pM on-plate loading concentration using Diffusion Loading mode and the Sequel Sequencing Plate 2.0 (Pacific Biosciences, USA).

### Sequence analysis

Circular consensus sequences (CCS) for each multiplexed sample were generated with the SMRT Analysis Software (Pacific Biosciences, USA) and used for further downstream analyses. An average of 7 Gb total output per SMRT cell was obtained, with an average CCS read length of 17 kb. Mean amplicon lengths of 1,500 bp were confirmed. For further sequence processing Mothur 1.37 was used^108^. From a total of 140,092 sequences, 58 sequences with homopolymer stretches of >8 were removed. There were no sequences containing ambiguous bases and further details of the sequencing output such as the average length, error rate and quality are summarized in Supplementary Table S2. Sequences were aligned using the align.seqs command in combination with the Silva v128_SSURef database. Reads that could not be aligned were removed and the remaining sequences were preclustered at 1% difference to account for potential PCR errors and then checked for chimeras using UCHIME in de novo mode^109^. Classification was done using classify.seqs using the RDP classifier implemented in Mothur^110^ and Silva v128_SSURef database^42,43^. Sequences classified as Chloroplast-Mitochondria-unknown-Archaea were removed from the dataset. Operational taxonomic unit (OTU) clustering was done with VSEARCH (dgc mode;^111^) as implemented in Mothur, using a 99% similarity cutoff to nearly represent one species per OTU. This cutoff was used to resolve relationships among closely related bacteria that would be masked when using a cutoff of 97%. Phylogenetic analyses were performed with the ARB software using the LTPs128_SSU tree^112^ and the SILVA database for bacterial 16S rRNA genes^42,43^.

### Phylogenetic analyses and statistics

Maximum-likelihood phylogenies (PhyML) were built with Jukes Cantor as the substitution model including 1,000 bootstrap replicates by using Geneious® 9.0.5^113^. To compare full-length with short read sequences, we restricted the sequences affiliated with the genus *Acinetobacter* using 16S rRNA primers to a 464 bp amplicon covering the hypervariable regions 3-4^41^. Beta-diversity analyses (i.e. box plots, PCoA and bar charts) were performed after rarefaction and log standardization of OTU-counts table using R version 3.5. and the package *vegan*. The differential abundance of OTUs in the inflow versus the effluent was computed on the non-rarefied OTU counts. The test used the exact negative binomial test in combination with the quantile-adjusted conditional maximum likelihood estimation of dispersion of the R package edgeR^114^. This analysis was based on TMM (trimmed mean of M values, where M is the log-fold-change of each OTU) normalized abundance data^115^. The test basically performed a pairwise comparison of OTU relative abundances between the two sample groups and an OTU was considered to respond significantly when the Bonferroni-corrected p-value was below 0.01.

## Supplementary information

Supplementary Information

## Data Availability

The dataset generated and analysed during the current study are available from the Sequence Read Archive (SRA) of NCBI (National Center for Biotechnology Information) under the BioProject ID PRJNA484334 and SRA accession SRP156296.
