# Supplementary material for: Characterization of bacterial communities in wastewater with enhanced taxonomic resolution by full-length 16S rRNA sequencing
Source: Sci Rep. 2019 Jul 4;9:9673. doi: 10.1038/s41598-019-46015-z (PMC6609626; doi:10.1038/s41598-019-46015-z)
Supplement: Supplementary file 1 — Supplementary Information [file 41598_2019_46015_MOESM1_ESM.pdf]

Supplementary information:

Characterization of bacterial communities in wastewater with enhanced taxonomic resolution by full-length 16S rRNA sequencing

Daniela Numberger, Lars Ganzert, Luca Zoccarato, Kristin Mühldorfer, Sascha Sauer, Hans-Peter Grossart, Alex D. Greenwood

Table S1: Comparison of the phylogenetic affiliation of *Acinetobacter*-OTUs between short (hypervariable region V3-V4) and full-length 16S rRNA sequence information. OTUs which could only be affiliated to a species when having full-length information are highlighted in bold.

	**short-length (477 bp)**			**full-lentgh (~ 1,500 bp)**		
	Affiliation	% similarity	Bootstrap	Affiliation	% similarity	Bootstrap
OTU003228	*A. junii*	99.0	39.4	*A. junii*	99.4	100
OTU004317	*A. lwoffii*	98.5	67.3	*A. lwoffii*	98.6	95.3
OTU004358	*A. harbinensis*	98.5	95.1	*A. harbinensis*	98.6	95.9
**OTU006694**	-	-	-	*A. celticus*	99.7	100
OTU011363	*A. ursingii*	99.0	99.8	*A. ursingii*	99.6	100
OTU012127	*A. albensis*	99.2	99.1	*A. albensis*	99.2	100
OTU012742	*A. baumannii*	98.7	94.6	*A. baumannii*	98.6	100
**OTU014283**	-	-	-	*A. oleivorans*	98.0	78.0
**OTU014486**	-	-	-	*A. beijerinckii*	99.7	98.7
OTU017554	*A. schindleri*	98.3	86.3	*A. schindleri*	97.9	98.7
OTU017557	*A. rudis*	96.7	87.9	*A. rudis*	99.3	100
OTU020140	*A. radioresistens*	98.5	94.9	*A. radioresistens*	99.1	100
**OTU020183**	-	-	-	*A. gandensis*	99.6	100
**OTU025639**	-	-	-	*A. indicus*	99.1	88.2
OTU025642	*A. gerneri*	96.2	69.5	*A. gerneri*	99.4	100
**OTU025938**	-	-	-	*A. haemolyticus*	99.1	77.5
OTU036224	*A. beijerinckii*	98.3	49.7	*A. tjernbergiae*	94.0	70.7
OTU037492	*A. haemolyticus*	97.3	66.3	*A. dispersus*	96.9	78.1

Table S2: PacBio sequencing statistics.

Total reads	140,092
Length average	1,492
Length range	1,402-1,623
Ambiguous bases	0
QC average	89.5
QC range	73-92
QC 50 bp sliding window	>89.6
Error rate average	2.90×10^{-9}
Error rate range	$5.3\times10^{-8} – 6.5\times10^{-10}$
Error rate 50 bp sliding window	1.10×10^{-9}
Homopolymers >8	58

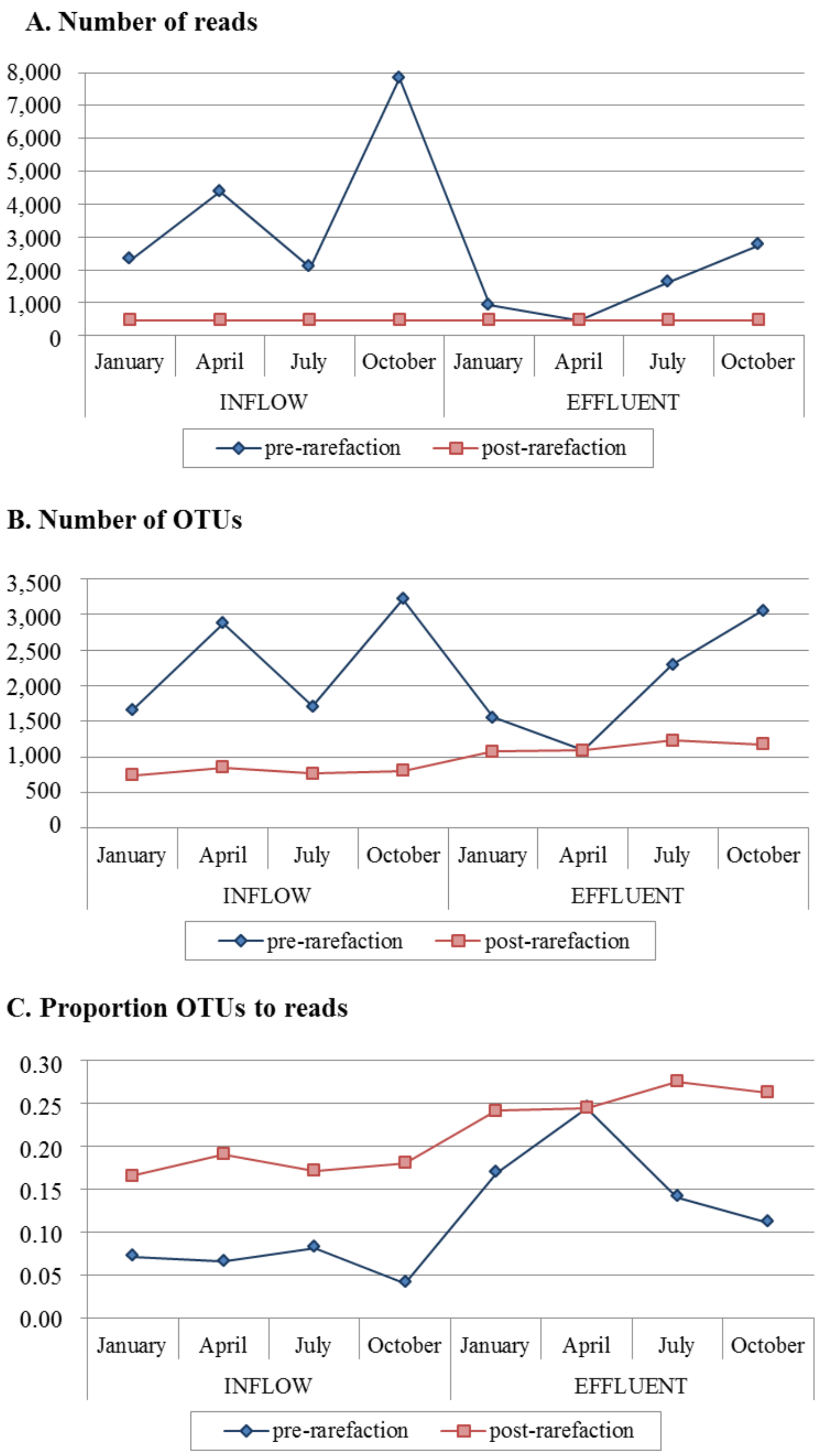

Supplementary Fig. S1: Differences in number of reads and OTUs between original and rarefied data. [A] Represents the number of reads pre-rarefaction (blue) and post-rarefaction (red). [B] Represents the number of OTUs pre-rarefaction (blue) and post-rarefaction (red). [C] The proportion of the number of OTUs to the number of reads pre-rarefaction (blue) and post-rarefaction (red) are shown.

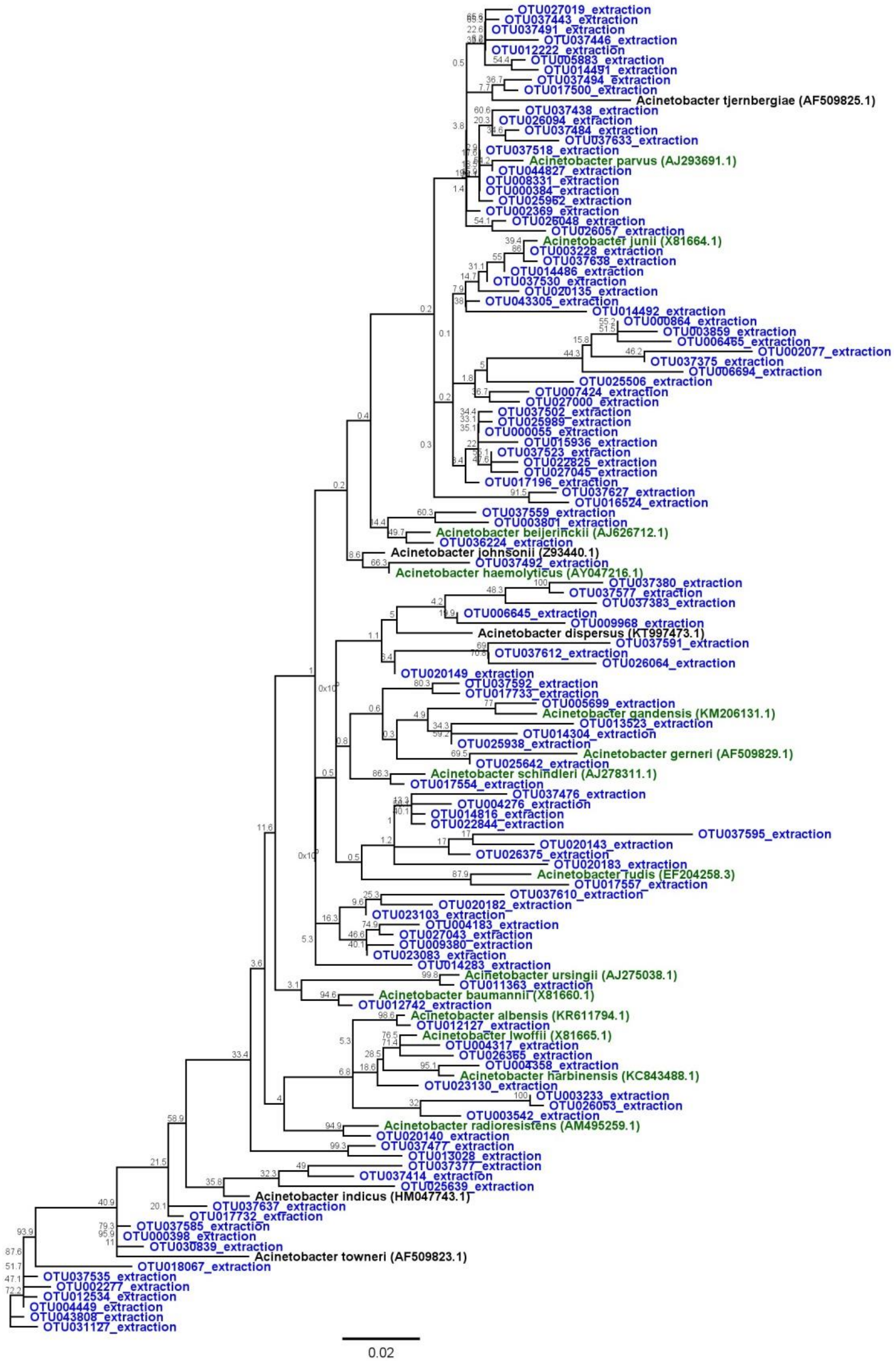

Supplementary Fig. S2: Phylogenetic tree of the genus *Acinetobacter* using 477 bp-sequences extracted from the whole 16S rRNA genes generated in the current study. Maximum-likelihood tree (PhyML), based on whole 16S rRNA gene or partial sequences over 1,000 bp. Bootstrap percentages at nodes are shown (based on 1,000 iterations). GenBank accession numbers are given in brackets. Bar represents 2% sequence divergence.

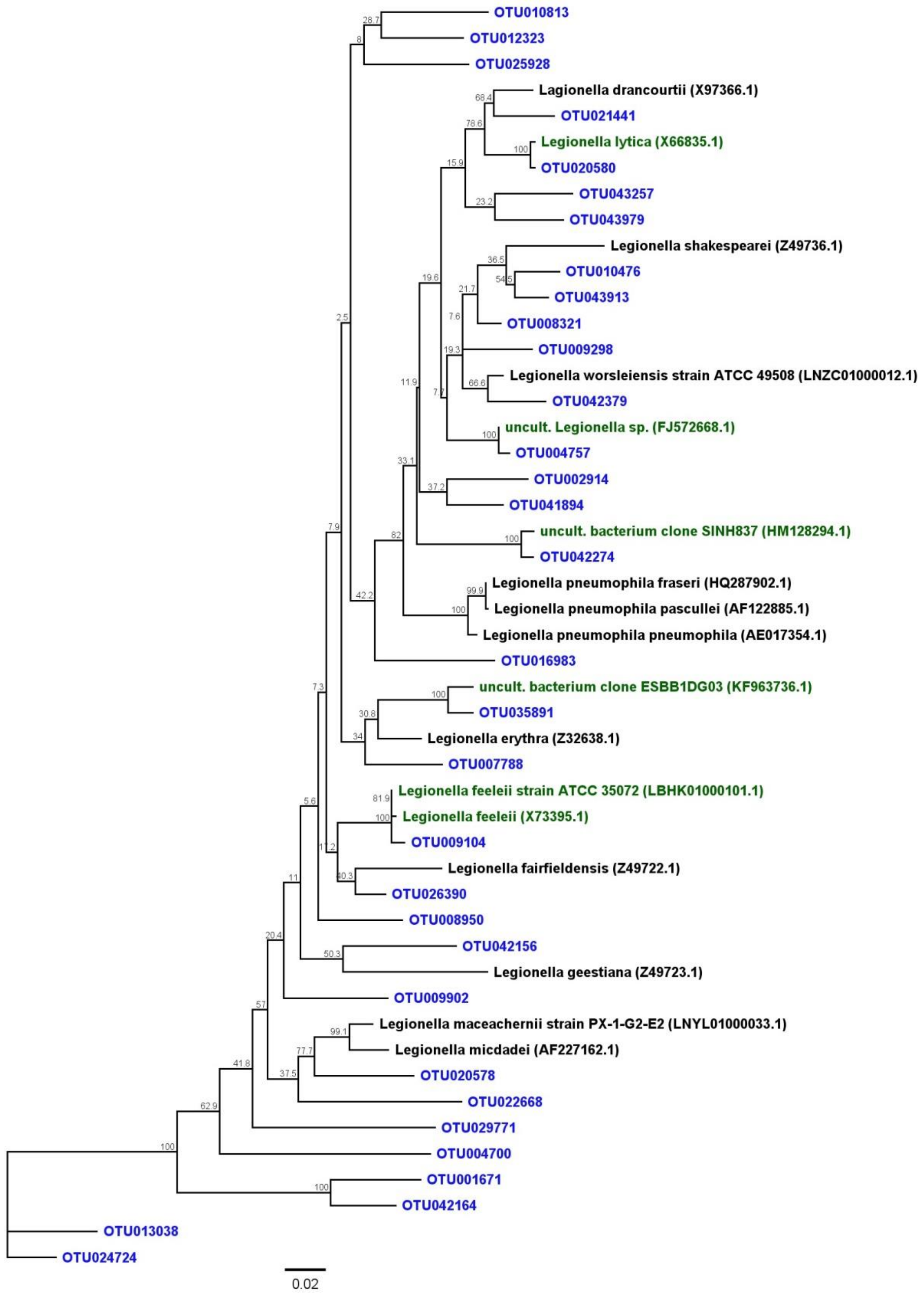

Supplementary Fig. S3: Phylogenetic tree of the genus *Legionella*. Maximum-likelihood tree (PhyML), based on whole 16S rRNA gene or partial sequences over 1,000 bp. Bootstrap percentages at nodes are shown (based on 1,000 iterations). GenBank accession numbers are given in brackets. Bar represents 2% sequence divergence.

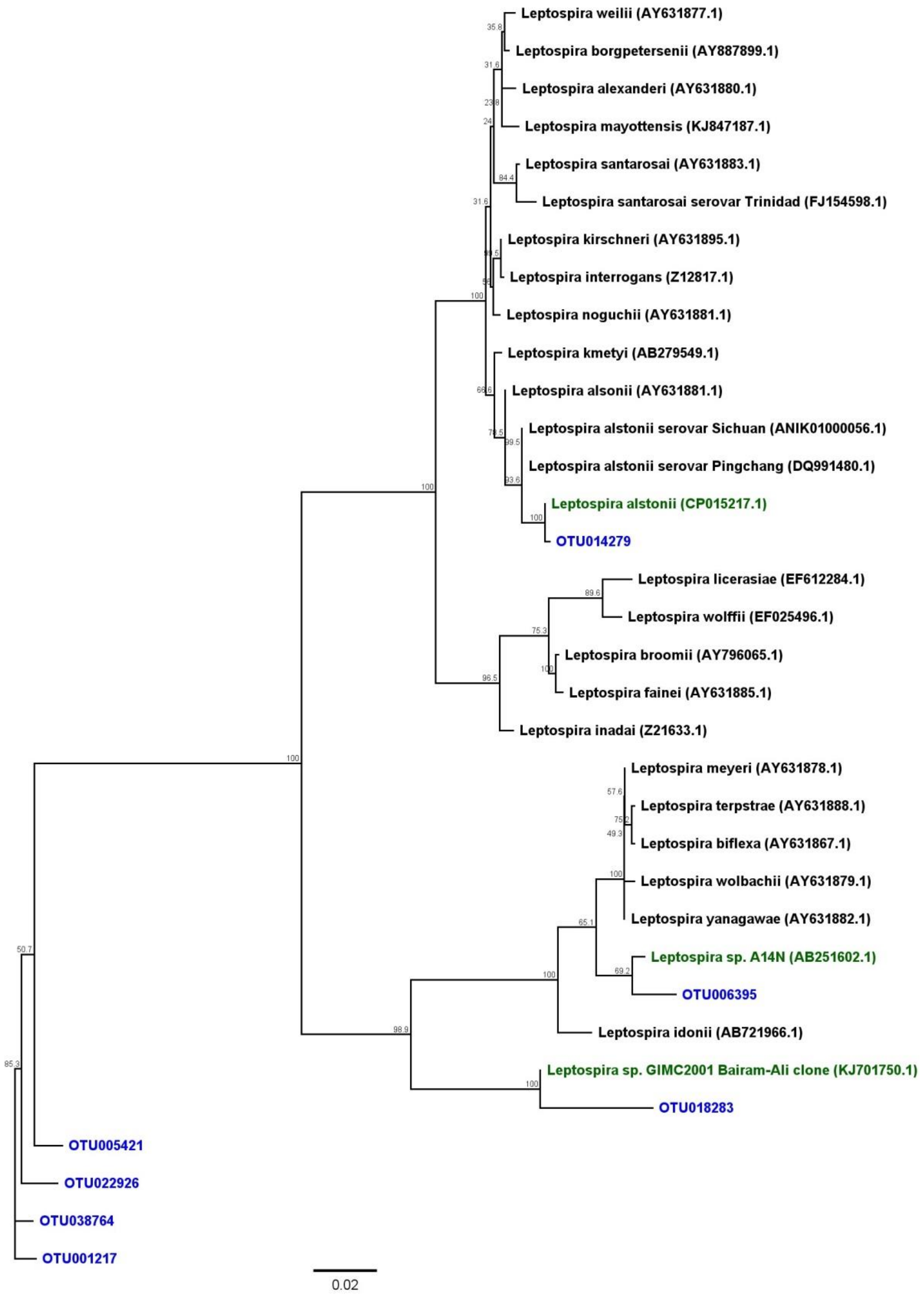

Supplementary Fig. S4: Phylogenetic tree of the genus *Leptospira*. Maximum-likelihood tree (PhyML), based on whole 16S rRNA gene or partial sequences over 1,000 bp. Bootstrap percentages at nodes are shown (based on 1,000 iterations). GenBank accession numbers are given in brackets. Bar represents 2% sequence divergence.

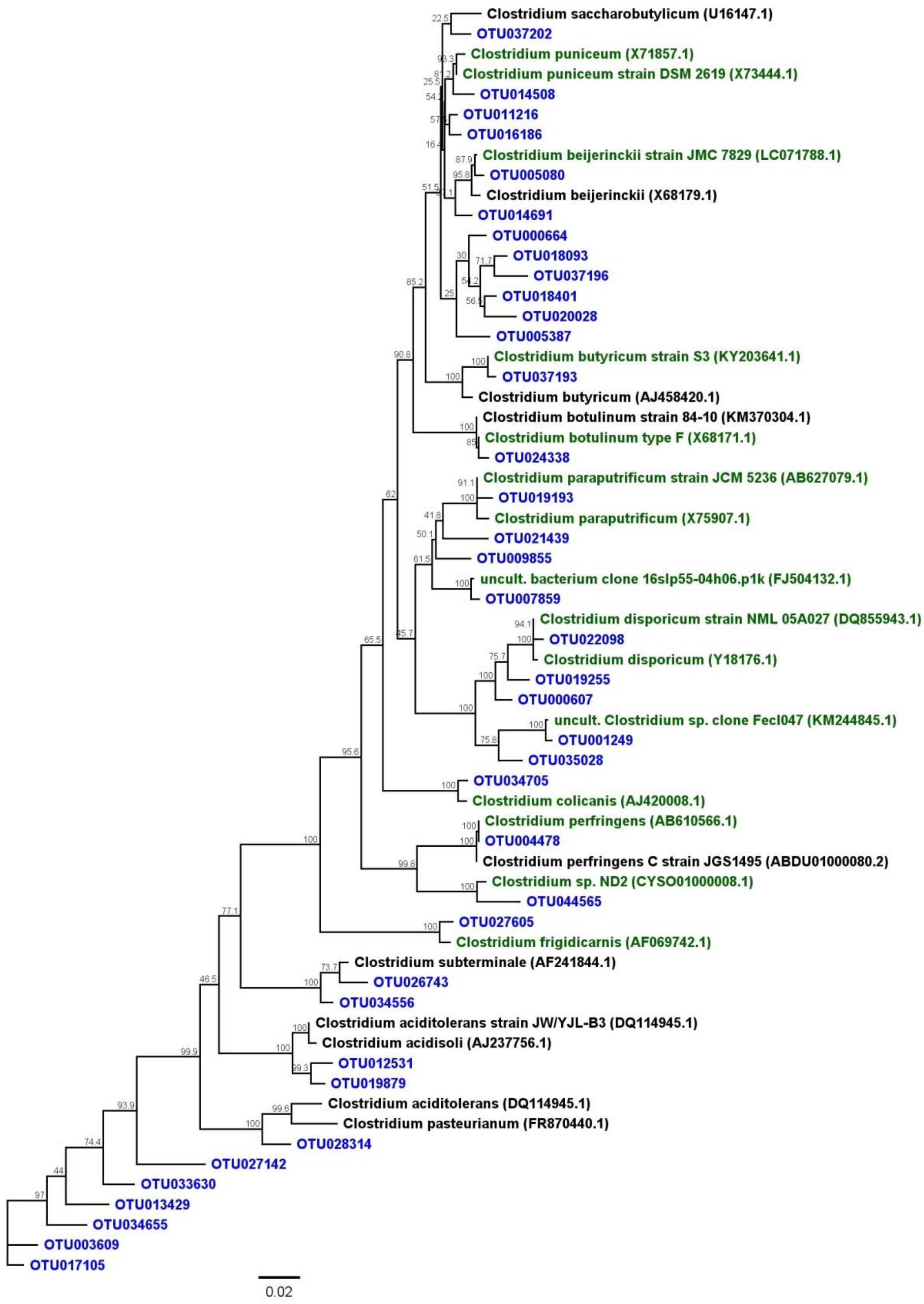

Supplementary Fig. S5: Phylogenetic tree of the genus *Clostridium* (sensu-stricto). Maximum-likelihood tree (PhyML), based on whole 16S rRNA gene or partial sequences over 1,000 bp. Bootstrap percentages at nodes are shown (based on 1,000 iterations). GenBank accession numbers are given in brackets. Bar represents 2% sequence divergence.

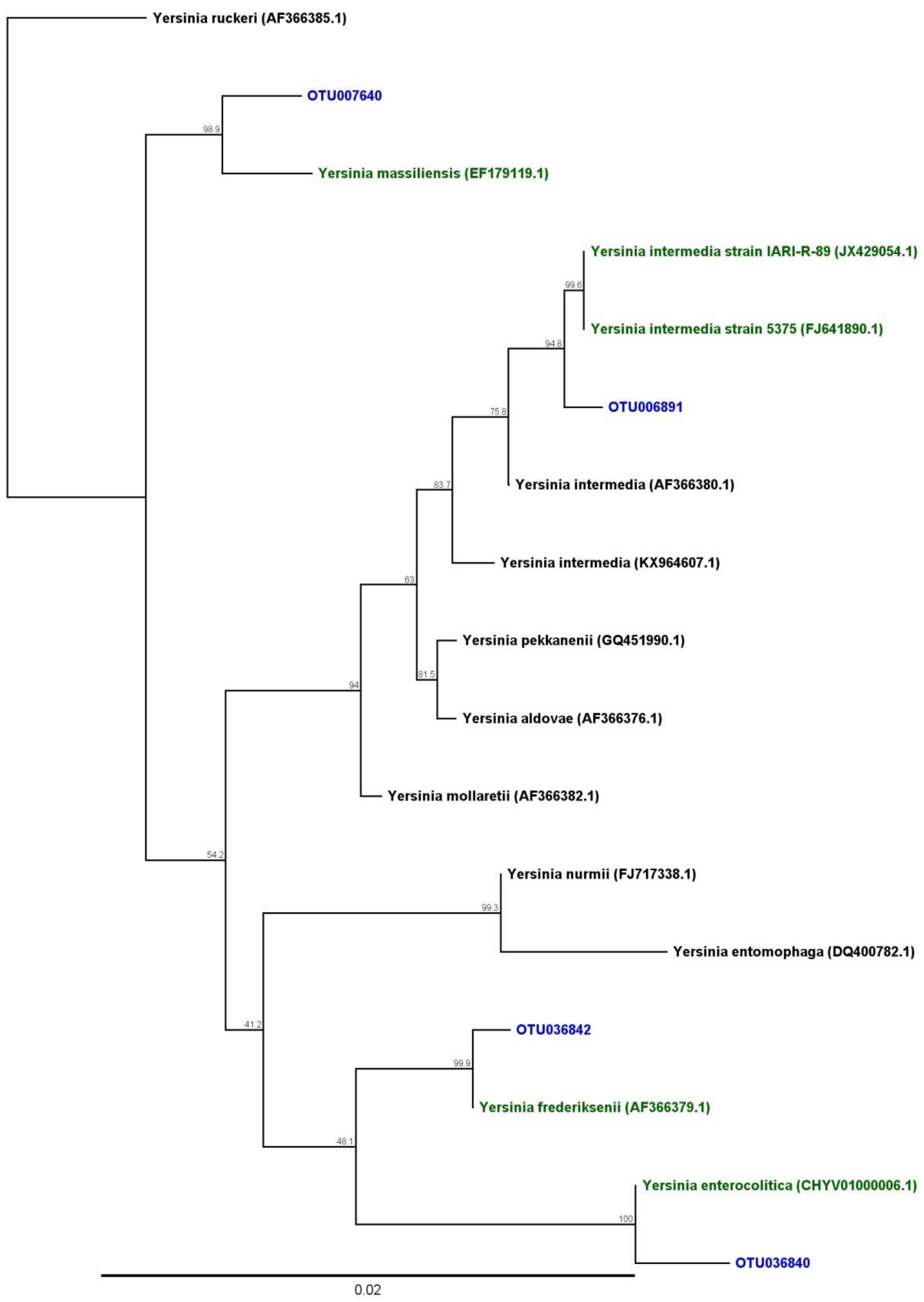

Supplementary Fig. S6: Phylogenetic tree of the genus *Yersinia*. Maximum-likelihood tree (PhyML), based on whole 16S rRNA gene or partial sequences over 1,000 bp. Bootstrap percentages at nodes are shown (based on 1,000 iterations). GenBank accession numbers are given in brackets. Bar represents 2% sequence divergence.

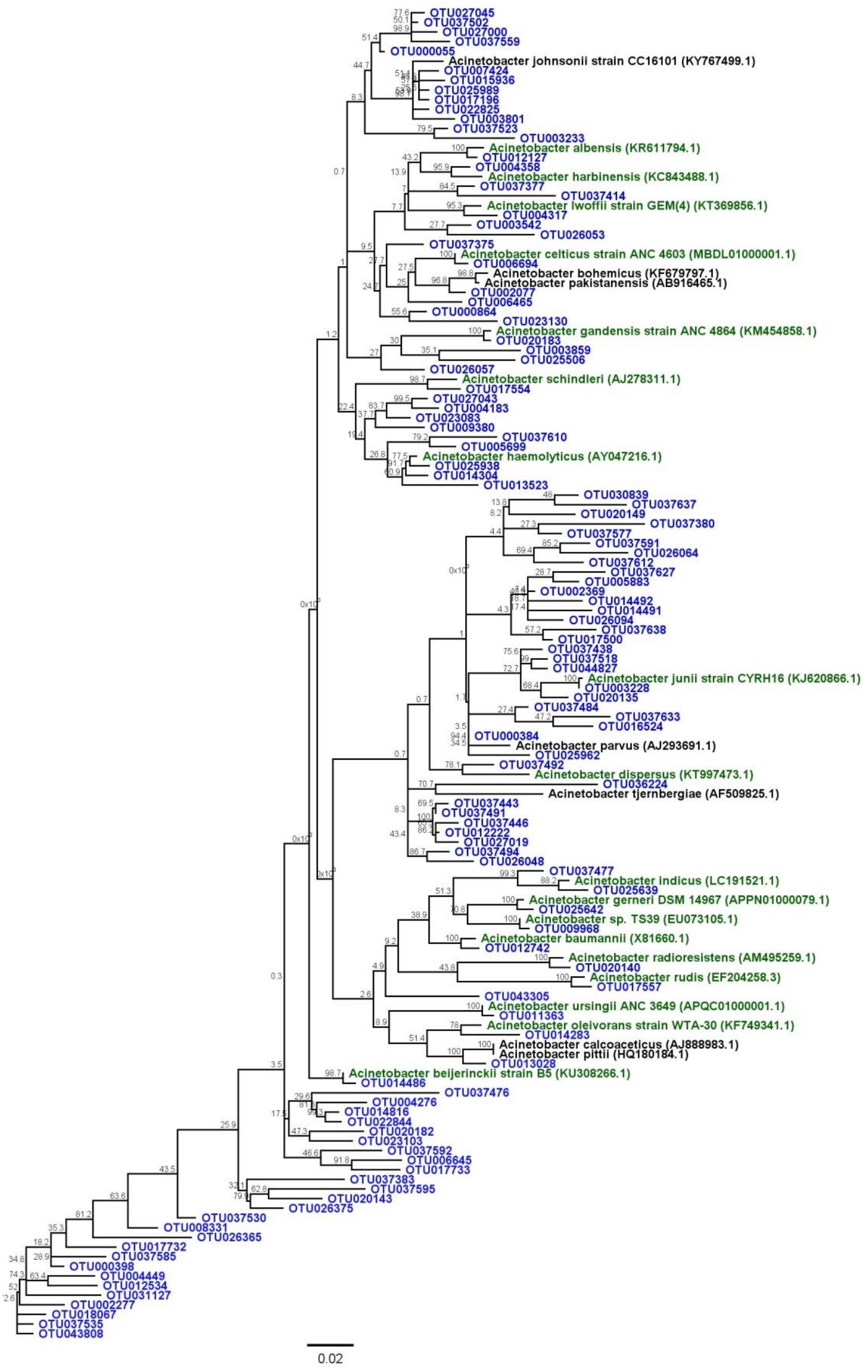

Supplementary Fig. S7: Phylogenetic tree of the genus *Acinetobacter*. Maximum-likelihood tree (PhyML), based on whole 16S rRNA gene or partial sequences over 1,000 bp. Bootstrap percentages at nodes are shown (based on 1,000 iterations). GenBank accession numbers are given in brackets. Bar represents 2% sequence divergence.

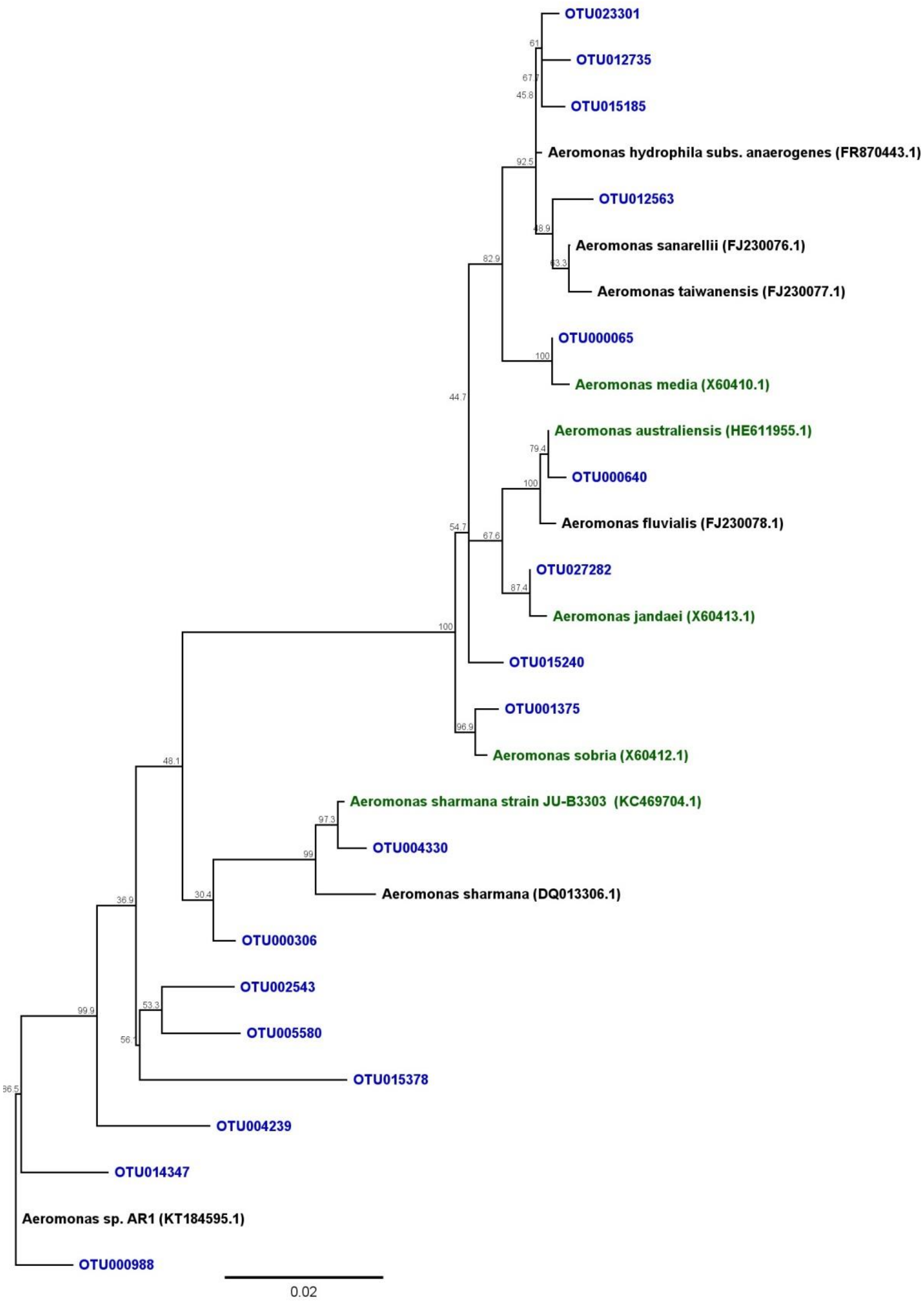

Supplementary Fig. S8: Phylogenetic tree of the genus *Aeromonas*. Maximum-likelihood tree (PhyML), based on whole 16S rRNA gene or partial sequences over 1,000 bp. Bootstrap percentages at nodes are shown (based on 1,000 iterations). GenBank accession numbers are given in brackets. Bar represents 2% sequence divergence.

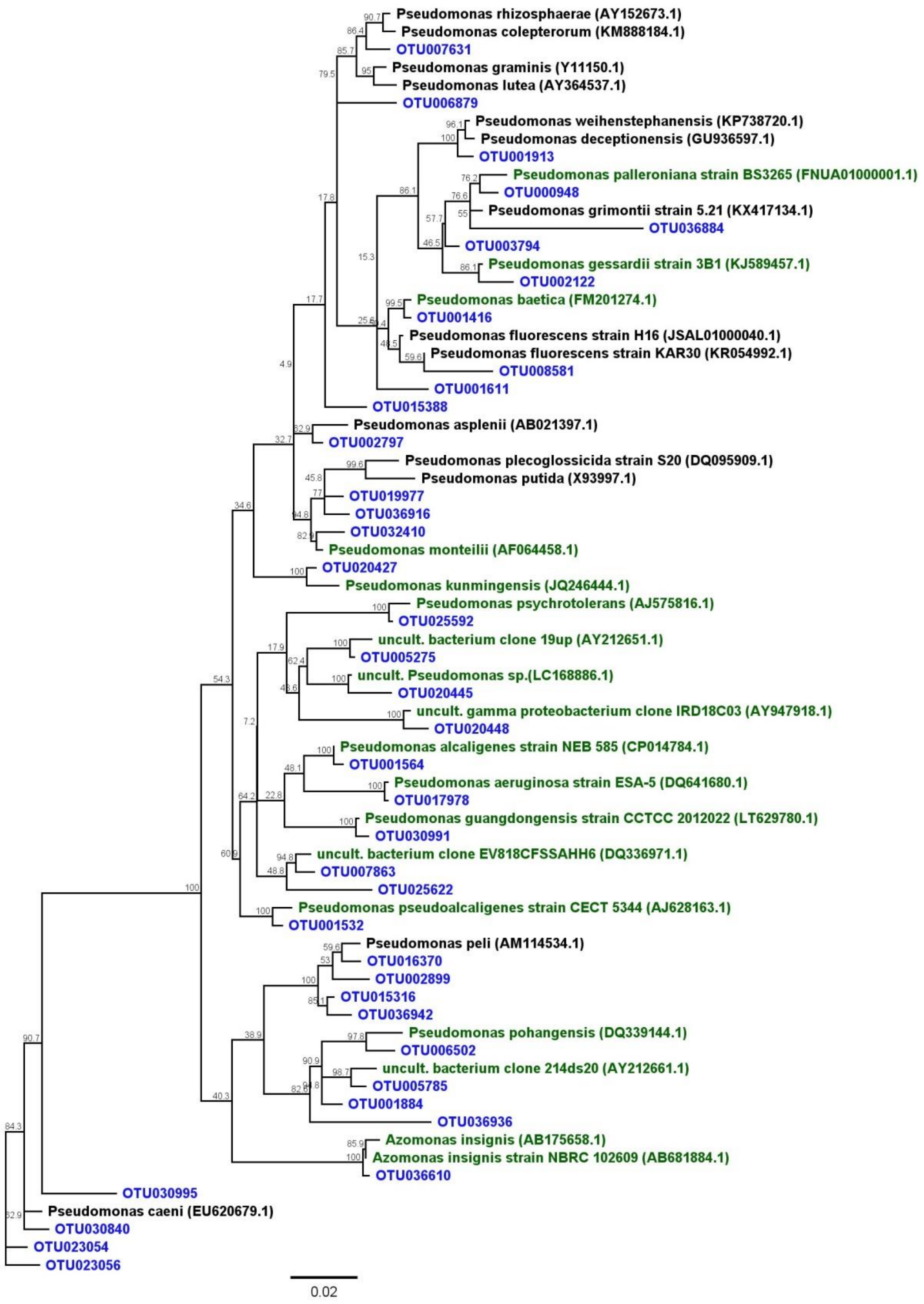

Supplementary Fig. S9: Phylogenetic tree of the genus *Pseudomonas*. Maximum-likelihood tree (PhyML), based on whole 16S rRNA gene or partial sequences over 1,000 bp. Bootstrap percentages at nodes are shown (based on 1,000 iterations). GenBank accession numbers are given in brackets. Bar represents 2% sequence divergence.

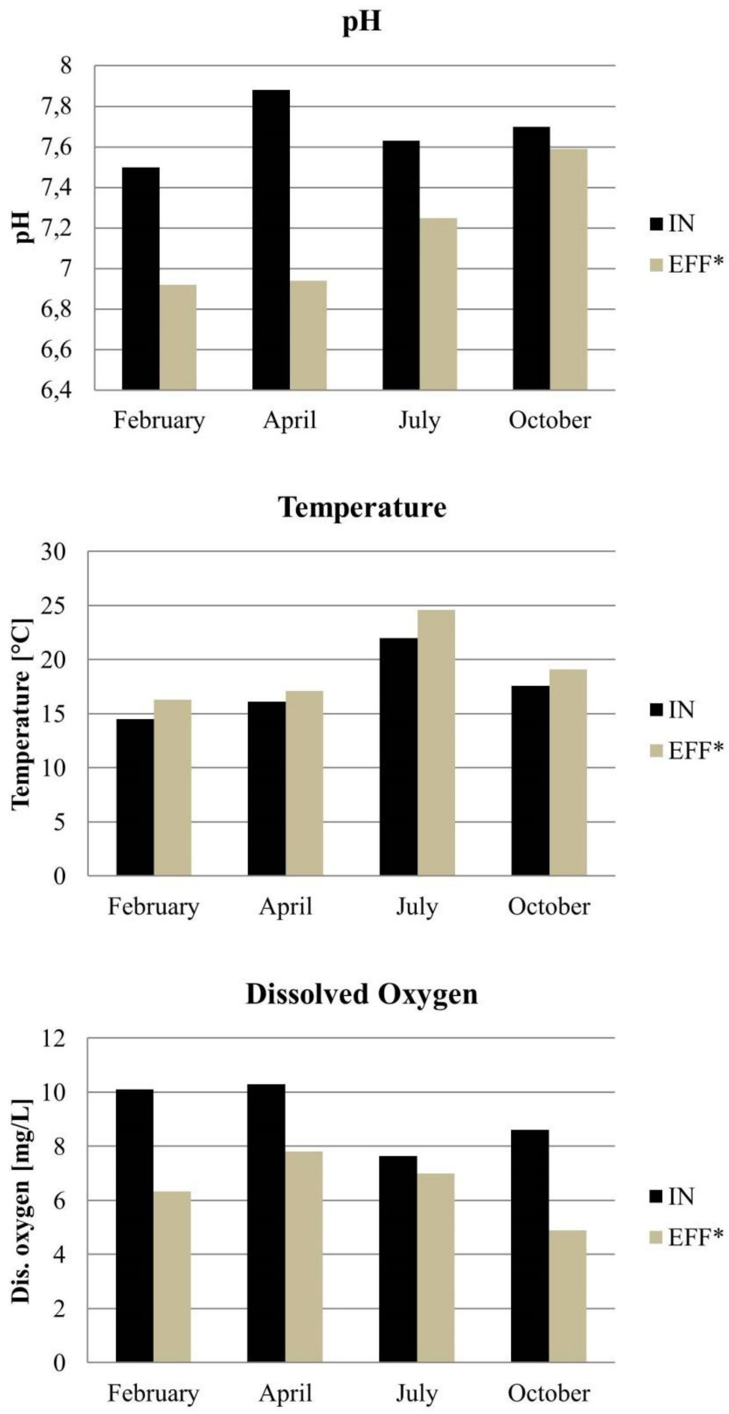

Supplementary Fig. S10: Measurements of pH, temperature and dissolved oxygen from inflow [IN] and effluent [EFF] samples. * Effluent samples were measured by the WWTP staff.
